# Supplementary material for: Older People’s Experiences of Living with, Responding to and Managing Sensory Loss
Source: Healthcare (Basel). 2021 Mar 15;9(3):329. doi: 10.3390/healthcare9030329 (PMC7998691; doi:10.3390/healthcare9030329)
Supplement: Supplementary file 1 [file healthcare-09-00329-s001.zip › Supplementary material 1- Interview guide.docx]

**Supplementary material 1: Interview Guide**

Interview objective

To investigate sensory impairment in community-dwelling adults 65 years and older, focusing on their recognition, experiences and management of self-identified sensory impairment.

Research questions

- To what extent is sensory change apparent to older people?
- Which of the senses is the most important to older people?
- What is the older person’s experience of living with sensory change?
- How do older people manage sensory change?

Equipment

- Two digital recorders
- Participant information statement and consent forms
- Notes and stationery

| **Research question and corresponding prompts** | **General aims of questions** | **Timing** |
| --- | --- | --- |
| Introduction   1. Introduce self and student investigator 2. Introduce the aim of the study    - This study is to understand your experiences of living with and managing sensory loss. This interview will explore some of your basic background demographic details, identify what sensory changes you have experienced and for how long, and obtain information about how you manage sensory changes.    - As stated in the Participant Information Statement, your identity will be kept confidential and any information you provide will not be used to identify you in any way. You also have the right to end this interview at any time you please and you also have the right to withdraw from this study at any point in time.    - This interview will take approximately 1 hour and 30 minutes and as explained in your consent form, I will audio-record this interview, with your permission.    - Do you have any questions about the study?   Respond to questions asked. | Reiterate information on the study and rights as participants of this study | 5 mins |
| Warm up questions   - How often do you attend the program/s in this centre? What do you think about it? - Do you live nearby?   Prompt questions:   - Besides attending the community program, what other activities do you regularly get involved in? | Explore participant’s everyday activities + build rapport | 5 mins |
| Background demographic information  Ask questions about the person’s age range, cultural background, language spoken at home, illness being treated, use of mobility aids, living situation (alone, with family, with others). | Identify background information | 10 mins |
| History of sensory change  We are really interested in changes that have occurred in your senses and your experiences living with them. By senses, we mean your sight, hearing, taste, touch, smell and feeling balanced. What changes have occurred in relation to any of these senses? Let us focus on one sense at a time.  Prompting questions:   - When did you first notice this change? - Were there any particular situations which made you realise that a change was occurring? - Was it a gradual change or a sudden change?   NOTE: If the participant has multiple sensory changes, we will discuss one sensation at a time. Sensation will be chosen based on question, “Which of the senses would you like to discuss now?”. | Explore the participant’s sensory change | 10 mins |
| Research Question 1: To what extent is a sensory change apparent to older people?  Have you ever noticed any changes in your other sensations besides the one you mentioned earlier (taste/touch/smell/vision/hearing)?  Prompt questions if only one sensory change is mentioned:   - Vision   - Have you noticed any changes in your vision recently? - Smell   - Do you notice any changes in your sense of smell? - Touch   - Have you ever cut yourself/sustained an injury and not realise it until later?   - How is your grip strength?   - How is your sense of balance? - Taste   - Do you notice any preference for sweeter food recently?   - Do you find yourself adding more salt/pepper into your food recently? - Hearing   - Have you noticed any changes in your hearing recently? | Explore the participant’s awareness to their other senses | 15 mins |
| Research Question 2: Which of the senses is the most important to older people?  Out of all your senses (sight, hearing, smell, touch and vision), which one is the most important to you? | Explore the most important sense to the participant | 5 mins |
| Research Question 3: What is the older person’s experience of living with sensory change?  Could you tell me a bit more about your experiences living with changes in sensation?  Prompt questions:   - How has the change in sensation impacted your life? - Has it impacted your safety? - What do your family members/those around you think of these sensory changes? - What adjustments have you had to make in living with sensory loss? - How do you feel about these life adjustments? | Explore the participant’s experiences living with sensory change | 20 mins |
| Research question 4: How do older people manage sensory change?  I’ve noticed you are wearing (e.g., glasses/hearing aids/corrective aids).  Prompt questions:   - Who do you currently receive help from in managing sensory changes? (eg: specialist medical and other services) - Do you have access to specialist medical and other services to manage sensory changes? - What factors influence your decision in seeking services/aids to manage your sensory changes? - To what extent does cost/finances influence your choice of management? - Do you need to pay for these specialist services? - How important are specialist services in managing your sensory changes? - If you are not able to pay for specialist services, how has this impacted on your management of sensory changes? - What do you do personally to manage this sensory change? (+ other relevant prompt questions, depending on responses)   We’re interested in whether you set goals for yourself when managing changes in your senses (e.g. sight, hearing, touch, smell, sense of balance).  Prompt questions:   - What do you focus on when managing your sensory change/s (e.g., sight…)? - How do you go about managing this change (e.g., sight...)? - What adjustments have you made in everyday day life to manage the change in your … (e.g., sight…)? - Who else is involved in managing changes in your … (e.g., sight…)? - Are there reasons why you have not chosen to obtain specialist services and aids in managing your sensory changes? - In managing your sensory change, what are the most important considerations for you in managing your sensory change/s? | Explore the participant’s management of sensory change and factors associated with satisfactory management of sensory change | 30 mins |
| Cool down   - Ask if participant if they have any questions or wish to stay anything else. - Thank the person for giving their time to answer the questions. - Remind the person that they are able to receive a summary copy of the study findings if they have ticked the relevant box on their consent form, and check if this is what they want. | Provide the person with an opportunity to contribute further information, and receive information about the study | 5 min |
