# Supplementary material for: Older People’s Experiences of Living with, Responding to and Managing Sensory Loss
Source: Healthcare (Basel). 2021 Mar 15;9(3):329. doi: 10.3390/healthcare9030329 (PMC7998691; doi:10.3390/healthcare9030329)
Supplement: Supplementary file 1 [file healthcare-09-00329-s001.zip › Supplementary material 2 - Data table.docx]

**Supplementary material 2: Data table**

| **Research question** | **Theme** | **Code** | **Excerpts (unique participant code)** |
| --- | --- | --- | --- |
| 1. To what extent is sensory change apparent to older people | Became apparent over time | Gradual change occurred | And then it’s got progressively, slowly, worse (I001_C1)  …it’s been gradual over the last 20 years. (I005_C4)  …it was alright then, it was pretty good then, then it started to deteriorate later in life now that it is more so now than what it was before. (I006_C4)  …it was gradual, it just started to get very very gradual very very slowly. (I011_P) |
|  |  | | |
|  | Not immediately apparent | Initially unaware of change | …if you don’t hear something, you don’t hear it, you don’t know you don’t hear it (I001_C1)  I haven’t noticed very much of what…. If there is change in my touch. I have not felt it. (I002_C1)  …you don’t realise what it is until you lose it (I007_C4) |
|  |  | Apparent when identified by family/carer | I only realised (hearing loss) when (husband) told me because he was very soft-spoken. (I002_C1)  Well just my daughter said, “Mum I think you are going deaf”. (I013_C5)  She’ll (carer) say, “You are talking very loud, talking very loud”. (I006_C4) |
|  |  | Apparent when identified by doctor | …it was noticed for me, when, when the Vietnam War was on and conscription was coming out they knocked me out because of deafness. (I004_C3)  …you don’t realise, you don’t have any symptoms really, suddenly, you got eye degeneration, it is age-related they say. (I007_C4) |
|  |  | Apparent when realising behaviour change | I’m shouting I can suddenly hear I’m shouting I’m sorry. (I001_C1)  I feel sometimes I am talking a bit loud… you know that I can hear myself that I talk a bit louder than like if I talk softer like that, but I don’t, I talk loud. (I006_C4) |
|  |  | | |
|  | Became apparent in a particular situation | Sudden awareness | I was having a very intimate intense conversation, I suddenly realised later I wasn’t sure whether I was saying yes or no at the right time. (I004_C3)  …it’s interactive business when you are a teacher like I am. I just couldn’t hear what they were saying…that’s when I first noticed it. (I001_C1) |
|  |  | Following treatment | I have terrible taste, at the moment especially because after I came out of hospital (post-chemotherapy), no taste in my tongue. (I008_C4) |
|  | | | |
| 2. Which of the senses is the most important to older people? | Eyesight is the most important | Difficult to fully function | I think I’d rather put up with anything else but my eyes last cause once you lose your eyesight you can’t do anything. (I009_C4)  It would be really difficult to… to get around without eyesight (I003_C3)  It’s one of the main things of life isn’t it, to see and all that. (I010_C4) |
|  | | | |
| 3. What is the older person’s experience of living with sensory loss? | Impacts on independence in activities of living | No longer able to drive | if I lose the license is terrible, I’ll just be…. housebound you know. (I007_C4)  I’ve lost my driver’s license, that’s a major…(I012_C4) |
|  |  | Difficult to shop | I can’t see the prices….terrible, I can still see and look what’s…., last time I had to ask somebody what it is. (I007_C4)  …couldn’t see what is this, you buy it, don’t know what it is (I007_C4)  I mostly ask someone else to read them (product details) while they’re there (at the grocery store). (I011_P) |
|  |  | Losing independence in living arrangements | I am happy living independently…because I can read and write. If I can’t do that then I become dependent. And my independence will go. (I002_C1) |
|  |  | Difficult to conduct business by phone | It is really really really irritating that companies, commercial companies want to phone you, they don’t want to email you, which means I am at the mercy of being able to understand whoever is on the phone, with typically little understanding of hearing impairments (I003_C3) |
|  |  | | |
|  | Impacts on established lifestyle | Reduces working opportunities | I had to give up lecturing even because to teach I ask questions, it’s interactive business when you are a teacher like I am. I just couldn’t hear what they were saying. (I001_C1)  I was an anthropologist really, critical to be able to hear and record things. I can no longer trust my abilities to do those things. (I001_C1)  …what annoys me is when the noise level gets too high and I can’t participate and …. I always use to sit in lecture theatres or in things and say well look I am not coming down the front like a dummy, you have to make sure there is a loudspeaker running around (I004_C3)  I used to be handyman…. I need, I use my fingers you know, doing things, my hands, I just can’t do it, can’t see it…. (I007_C4)  (Vision changes) promoted it (retirement). I mean I would have probably retired anyway but it forced, forced the issue (I012_C4) |
|  |  | Reduces engagement in leisure activities | I’ve given up book reading and that’s a huge loss…I live up in this library with books. That’s the saddest thoughts. (I001_C1)  I’m interested in reading and all those things, I can’t do that all the time, my eyes are not good anymore so… (I002_C1)  I’m having trouble reading and I’d just like to make sure that uh I can keep on reading or seeing the pictures on the TV and all that. So, that’s important to me. (I010_C4)  …can’t play golf anymore, can’t……too easy to lose my balance (I012_C4)  I really can’t understand everything that and in church sermons if they speak very quickly, I don’t really know what they are saying (I013_C5) |
|  |  | Challenges self-identity | …that’s life….nothing lasts forever, everyone says you look young, you look….I wish I could feel the feeling (I007_C4)  Well not really, well, what what can you ask for them to do for you? Take 5 years off your age? (I005_C4)  I wished I was 21…. I’ll settle for 22, 27! (I003_C3) |
|  |  | | |
|  | Impacts on social life | Avoiding social interaction | I avoid bigger groups it is chaos (I001_C1)  I avoid all kinds, I avoid gatherings and that’s bad for someone…who is used to large groups. (I001_C1)  I don’t mind living in silence, and I can find a silence beyond deafness. (I004_C3)  I don’t talk when I am in the van with the others because if they can’t hear me and I can’t hear them, you know then we will be making a mess, so I say it is best to be quiet. (I008_C4) |
|  |  | | |
|  | Taking measures to retain self-determination | Making no immediate changes to address impairment | “Your left hearing is getting, is going down” and I wasn’t so bothered then because I had my right ear and my right ear was very good. (I008_C4)  …the doctor said when you gave me the story, you better get your affairs in order which sort of……..um he probably tells everyone to get their affair in order at the magic age and um……so I didn’t take much notice of it until I made another fall, minor but showed that it (my vision) wasn’t anything better than before. (I012_C4) |
|  |  | Changing behaviour to accommodate impairment | No, I just you know um wave my eyes around (when I get split vision), no but it doesn’t really bother me it often happens at home. (I005_C4)  I don’t know…. When I…. just when I see things I have, I have no problem looking at things but I have to widen my eyes um… yeah (I009_C4) |
|  |  | Choosing not to use aids | Outside, when I get out of the place, out of my unit, I don’t wear hearing aids. Even now I wasn’t wearing it, I just brought it with me. (I002_C1)  Otherwise, when nobody is coming, I don’t wear it. Even when I go out for a walk I don’t wear it. Even when I have to buy some groceries, I don’t wear it. (I002_C1)  I don’t wear, I can’t stand it, I got it but I can’t. (I007_C4)  …but there is nothing they can do for you either unless they give you hearing aid….like I can’t have it, I can’t stand it. (I007_C4)  I don’t know, I don’t think they are very useful, I don’t know. (I009_C4) |
|  |  | Taking safety risks | I find my way (to the toilet) in the dark. (I001_C1)  I’m a bit worried (when going on night walks) but I go where there is streetlights. (I004_C3)  My house is a lot of clutter but in a way it is good because I can put my hand here, my other hand here and walk, walk around. (I007_C4)  Otherwise, I normally prefer not to (use my walking stick) because I … if I have to buy something then I can’t carry it if I have my walking stick to hold. (I002_C1) |
|  |  | | |
|  | Impacts on emotions and emotional responses | Causes personal annoyance and irritation | …what annoys me is when the noise level gets too high and I can’t participate. (I004_C3)  …they don’t look at me or mah-jong I couldn’t hear when they say pong, I had to stop because that makes me irritated and cranky. (I001_C1)  if I am wearing it (hearing aids), I can’t stand the loud noises at all. It gives me headache (I002_C1)  I was hearing all kinds of things that I would quite happily not hear you know, the rustling of paper and all that stuff. (I003_C2)  I got the hearing aid but I can’t stand it. Makes everything louder you know like, the background noise, like paper, that noise is in your hearing aid, that’s terrible, paper, paper noises, making noises with paper, I just can’t stand it. (I007_C4) |
|  |  | Causes strain within the family | What happens is the kids say, mum I asked you, mum wear the bloody, mum for god’s sake, and they can’t, you can’t hear them and they get cross. (I001_C1)  Initially the family are irritated right, because they don’t understand. (I001_C1)  …not so nice with my brother because he is impatient and has perfect hearing. (I001_C1)  I feel like I’ve been ostracized (by my family) because I am not hearing everything that is being said. (I010_C4) |
|  |  | Become resigned to the impairment | I try to put it (TV) up louder sometimes, and other times I think I just don’t mind, I just…. accept it. (I009_C4)  I think everyone else know what’s going on but me, so that’s it, that’s you know I think oh well, I’ll hear most probably later on. (I006_C4)  you don’t know what you have when you have good sight you know….you don’t realise what you have…you only realise after you lost it, then you see what it is…but….that’s how it is. (I007_C4)  you’re just a bit slower than you used to be, get used to it. What can you do about it. (I005_C4)  I think it is important but same time I don’t think they can do anything for me. Just not to be able to hear somebody on television…(I009_C4)  I can’t expect much more at my age. (I005_C4)  I can’t do anything about it….as much as I can try. (I007_C4)  I need to now but I haven’t been to a specialist for a while. So I haven’t been…I just…. Can’t be bothered attitude. (I009_C4)  I live from day to day and that’s it, that’s all I can do now, can’t do anything more…. (I006_C4)  I don’t, I don’t set any goals, I just take it day by day as it comes. (I011_P) |
|  |  | Feel helpless to improve the situation | Of course I feel helpless, awful… it is not easy….some people accept things easier, some people are… not you know (I007_C4)  I feel awful...afraid I might get blind you know, how am I going to live (I007_C4)  What can they do? Can you help me with my eyesight? No, I can’t do anything about it (I007_C4)  Well I would like to hear better, I am not satisfied but I’ve tried to improve and it didn’t so what can I do. (I003_C2) |
|  |  | Feel disabled with continued impairment | …disabled if I can’t read or see you know what I need to read or see. (I003_C2)  I would be lost again without glasses (I003_C2)  It is something I got to have all the time you know cause I can’t hear without it, I can’t hear very much without it (I006_C4) |
|  |  | | |
|  | Increased dependence on family members | Reliance on family members for medication support | my daughter-in-law gets them (medications) ready for me and uh I got to have them in the morning and at night before I go to bed (I010_C4) |
|  |  | Reliance on family members for transport | I can’t get my daughter or son-in-law to anytime take me there (to the specialist), only when there are not working time (I010_C4) |
|  |  | Reliance on family members for emotional support | …talk to my brother last night…, because I wanted a source of energy. (I004_C3)  …my daughter is there, she is always giving me strength and all that….. (I008_C4) |
|  | | | |
| 4. How do older people manage sensory loss? | Obtain medical and technical support | Use sensory aids | if I didn’t have hearing aids I wouldn’t be able to hear you. (I004_C3)  It is something I got to have all the time you know cause I can’t hear without it, I can’t hear very much without it. (I006_C4)  I’d be lost without them (glasses). So yes they help a lot. (I003_C2) |
|  |  | Have sensory check-ups | The optometrist, I see regularly. (I001_C1)  I go (for eye check-ups) annually. (I013_C5)  That’s what, the optician or whatever it is he gives me a test each year. (I004_C3)  Ophthalmologist I think it is, the eyesight, I get called in there once a year to check out my eyesight. (I004_C3)  He, he (audiologist) will ring to remind me, if he thought, it was being a bit long. (I001_C1)  When I feel like these (hearing aids) need to be cleaned or serviced, my aids, then I have to go. (I002_C1)  I go (to the audiologist) when I need to when I sort of feel like emergency, but I usually stay away and just go when they ask for appointments. (I004_C3) |
|  |  | | |
|  | Adapt to sensory changes | Accept the changes occurring | No (my hearing has not impacted my life). I just try to make the most of it, I get around you know. (I004_C3)  You don’t get your hearing back. And that takes some getting used to. (I001_C1)  I don’t try to deny the fact that I can’t hear. (I002_C1)  I live with reality. This is my reality. (I002_C1)  What I’ve never had, never, is a fear of losing my hearing because it is in the family... So I knew it was likely to come so not frightened okay. (I001_C1)  I expect… we expect in our family though for at least 2 out of 3 of us it might be more than just a little bit. (I001_C1)  …deafness is in the family and uh I just have to live with that. (I004_C3) |
|  |  | Advocate for self and others with sensory impairment | I’ve always worked against uh, being invalidated by it. (I004_C3)  Encourage both those places to turn the loop on and to use a microphone for questions because after the lectures some of the questions are very interesting but I can’t hear them. (I001_C1)  I got little cards which say I have a hearing loss, please respond very carefully to what I need. (I004_C3)  I just make a point of it so that not only assist me, it also assists other people to make a check and be careful with deaf people. (I004_C3) |
|  |  | Develop new social roles | …that is where a lot of my energy has been directed (Research into sensory aids). Energies that used to go into full time job and teaching and so on. (I001_C1)  I have occupied myself with welfare works, with NGOs. (I002_C1)  I do occupy my stuff with a lot of ministry (pastoral care). (I004_C3) |
|  |  | Maintain social and family relationships | (Day centre) it’s taken my mind (off the sensory changes) … thinking different things, you meet other people and talk (I007_C4)  …talk to my brother last night…, because I wanted a source of energy. (I004_C3) |
|  |  | | |
|  | Practice safe behaviour | Reduce environmental risks | When I first moved in I made sure I had a light switch at the bed. (I003_C2)  I always look you know I always stop and look (as I can’t hear the traffic). (I005_C4)  I’ve always you know, I was looking around on my left side to see if anything is coming (cause I can’t hear from my left ear) or you know when anything and my son has always take hold of my arm and that’s it. (I005_C4)  I keep a walking stick, handy for the time when I may need it, I am aware that I might need it. (I001_C1)  I am aware I must not stand on chairs, I must not change lightbulb, no matter how much it needs done because of falling. (I001_C1) |
|  |  | Proactively seek medical review | I want to see my specialist, I hope to get a booking for him, because I think my eyesight is going a little bit….the other way and I’d just like to make sure it is alright. (I010_C4)  I try to see my eyesight doctor in the last fortnight but I have no luck yet, but I hope so because I don’t want it to deteriorate anymore yeah. (I010_C4)  …it’s if anything else starts to go wrong, I go and see about it you know. If I start having problems with my eyes or my legs or anything like that. (I006_C4) |
|  |  | | |
|  | Lacking motivation to improve sensory impairment | Unable to afford further treatment | I was referred to a specialist, but I am having second thoughts about going with him because he is charging enormously for things that could be done for free. (I003_C2)  I couldn’t afford to truly, $400 to go and see someone for nothing much at all that you can get at a public clinic, but anyway. (I013_C5) |
|  |  | Not interested in making the effort | … seems to be alright. You get lazy. (I005_C4)  You see my glasses, I don’t think it is that bad and I never had me eye disease or problems or anything like that, and you get by the way things are… (I005_C4)  Well unless my hearing really went awry but I you know I just think it is just wax in the ear anyway (I009_C4) |
|  |  | | |
|  | Motivated to maintain/improve the senses | Undertake physical fitness to maintain the senses | I think the big thing was the gym, I used to go there 4 or 5 years at least and that was basically...the...foundation for most of your senses (I005_C4)  I work on that (exercise), and I know that takes, automatically takes care of some things…I do pay attention to it (exercise) and I don’t have to worry about touch and smell and whatever the other is (I001_C1) |
|  |  | Participate in sensory research | And so the acoustic engineers need subjects, human subjects, those who can fully hear and especially those who are using…. So I do that regularly, I’ve done that for about 6 to 8 years and through that I have learned a lot about acoustic engineering and so on and that is interesting (I001_C1) |
