# Supplementary material for: Older People’s Experiences of Living with, Responding to and Managing Sensory Loss
Source: Healthcare (Basel). 2021 Mar 15;9(3):329. doi: 10.3390/healthcare9030329 (PMC7998691; doi:10.3390/healthcare9030329)
Supplement: Supplementary file 1 [file healthcare-09-00329-s001.zip › Supplementary material 4 - Participant characteristics.docx]

**Supplementary material 4: Participant characteristics**

| **Unique identifier code** | **Age** | **Gender** | **Cultural background** | **Living situation** | **Glasses** | **Hearing aids** | **Use of mobility aids** | **Sensory change identified** |
| --- | --- | --- | --- | --- | --- | --- | --- | --- |
| I001_C1 | 82 | F | Australian | Alone | Yes ^a^ | Yes | Walking stick | Sight  Hearing  Touch |
| I002_C1 | 92 | F | Indian | Alone | Yes ^b^ | Yes ^d^ | Walking stick | Sight  Hearing  Taste |
| I003_C2 | 77 | M | Australian | Alone | Yes ^c^ | No | None | Sight  Hearing |
| I004_C3 | 69 | M | Australian | Alone | Yes ^a^ | Yes | Walking stick | Sight  Hearing |
| I005_C4 | 93 | M | English | Alone | Yes ^b^ | Not applicable | None | Sight  Smell  Taste |
| I006_C4 | 85 | F | Australian | Alone | No | Yes | Walking stick  Four-wheel walker | Sight  Hearing |
| I007_C4 | 94 | M | Slovenian | Alone | No | No ^e^ | Walking stick  Four-wheel walker | Sight  Hearing  Touch |
| I008_C4 | 89 | F | Indian | Alone | Yes ^a^ | No | Four-wheel walker | Sight  Hearing  Taste |
| I009_C4 | 85 | F | Australian | Alone | Yes ^b^ | No ^e^ | None | Sight  Hearing |
| I010_C4 | 93 | M | Australian | With family | Yes ^a^ | Yes | Walking stick | Sight  Hearing |
| I011_P | 76 | F | Australian | Alone | Yes ^b^ | Not applicable | None | Sight |
| I012_C4 | 77 | M | Australian | With wife | Yes ^b^ | No | Walking stick | Sight  Hearing |
| I013_C5 | 84 | F | English | Alone | Yes ^b^ | No | Four-wheel walker | Sight  Hearing  Smell  Taste |

(^a^: wears glasses daily, ^b^: wears glasses only for certain purposes (e.g.: reading, driving, outdoor activities), ^c^: wears different pairs of glasses for different purposes, ^d^: wears hearing aids only during direct conversations, ^e^: has hearing aids but chooses not to wear them).
